# PCA-Assisted Raman Analysis of Osteonecrotic Human Femoral Heads

**DOI:** 10.3390/mps5010010

**Published:** 2022-01-17

**Authors:** Eiji Ishimura, Wenliang Zhu, Elia Marin, Taigi Honma, Nobuhiko Sugano, Wataru Ando, Giuseppe Pezzotti

**Affiliations:** 1 Ceramic Physics Laboratory, Kyoto Institute of Technology, Sakyo-ku, Matsugasaki, Kyoto 606-8585, Japan; wlzhu@kit.ac.jp (W.Z.); elia-marin@kit.ac.jp (E.M.); taigihonma0530@gmail.com (T.H.); 2 Kyoto Municipal Science Center for Youth, Kyoto City Board of Education, Fushimi-ku, Fukakusa, Kyoto 612-1601, Japan; 3 Department of Dental Medicine, Graduate School of Medical Science, Kyoto Prefectural University of Medicine, Kamigyo-ku, Kyoto 602-8566, Japan; 4 Department of Orthopaedic Medical Engineering, Osaka University Graduate School of Medicine, Suita, Osaka 565-0871, Japan; n-sugano@umin.net (N.S.); w-ando@umin.ac.jp (W.A.); 5 Department of Immunology, Graduate School of Medical Science, Kyoto Prefectural University of Medicine Kamigyo-ku, 465 Kajii-cho, Kawaramachi dori, Kyoto 602-0841, Japan; 6 The Center for Advanced Medical Engineering and Informatics, Osaka University, Yamadaoka, Suita, Osaka 565-0871, Japan; 7 Department of Orthopedic Surgery, Tokyo Medical University, 6-7-1 Nishi-Shinjuku, Shinjuku-ku, Tokyo 160-0023, Japan

**Keywords:** osteonecrosis of the femoral head (ONFH), bone tissue, Raman spectroscopy, PCA, alcohol, steroid

## Abstract

Osteonecrosis of the femoral head (ONFH) occurs frequently in adolescents and young adults and causes progressive deformation and destruction of the hip joint and impairs standing and walking, resulting in a significant decrease in the quality of life of patients. In addition, studies have shown that a history of corticosteroid administration and heavy alcohol consumption are closely related to the occurrence of ONFH. However, the detailed mechanism by which steroid administration and alcohol consumption are associated with the development of the disease is still unknown. With many researches still ongoing and without a clear biological pathway for osteonecrosis, effective preventive measures cannot be taken. Therefore, the current focus of ONFH treatment is to establish an early diagnosis and treatment strategy. We obtained the femoral heads of four patients with steroidal ONFH and three patients with alcoholic ONFH. We then compared the femoral heads of steroidal and alcoholic osteonecrosis by analyzing them at the molecular level by Raman spectroscopy. Crystallographic changes (deformations) in the mineral phase and fraction of organic material respect to the total mass were then plotted as a function. We found that changes in bone composition in ONFH were different in steroidal and alcoholic ONFH. We conclude that this suggests that the developmental mechanisms of steroidal and alcoholic ONFH may follow different paths. We also noticed that while steroid seem to lead to a more marked degradation of the tissue, alcohol seem to affect also the quality of the healthy tissue.

## 1. Introduction

Osteonecrosis of the femoral head (ONFH) has been reported to be a common cause of acute hip pain in the early stage, following the progressive collapse of the femoral head with joint destruction [1]. The corrected annual prevalence of ONFH per 100,000 was 18.2–19.2 and peak distribution was observed at ages the 40s and 60s in males and females, respectively [2]. Among risk factors, alcohol- and steroid-associated ONFH had the highest prevalence of 47.0% in males and 49.8% in females, respectively [3]. Restoration of extensive necrosis requires surgical resection: in such cases, total hip arthroplasty (THA) is required, but this surgery is a highly intrusive one for the patient [4]. 

In ONFH, the bone tissue becomes necrotic due to impaired blood flow or other circulatory problems, which can be associated with, alcoholism, steroid ingestion, and smoking [5,6]. It is a serious condition that often impairs weight-bearing joints, such as the hip and knees. The hip joint, in particular, is the most commonly affected. Most patients require a total hip replacement after the femoral head loses its load-bearing function.

Some studies have evaluated survival and risk factors for progression and transition to THA, for example after porous tantalum implant surgery for the treatment of ONFH [7]. However, so far the chemical mechanisms behind ONFH are still mostly unknown. One study aimed at determining and improving the reliability of a classification system for ONFH [8], but no classification system so far could be clearly correlated with the origin of the disease. In Osteonecrosis of the jaw (ONJ) imaging could help identify patients at an early or preclinical stage, leading to an approach to prevent the disease [9], but this approach cannot be easily extended to joints. 

Recently, Raman spectroscopy has proven to be a powerful technique for studying biological tissues [10]. Either by using advanced techniques as surface-enhanced Raman spectroscopy (SERS) [11] or by carefully tuning conventional high-resolution spectrometers [12], the information extracted by Raman spectroscopy resulted to be comparable with more conventional diagnostic techniques and has shown the advantages of its superior spatial resolution and broad detection capability [13].

As previously shown [14], Raman spectroscopy, with its excellent spatial resolution and high-resolution measurement capability, can be a powerful tool for studying bone chemistry and may be useful in future studies on bone metabolism during the evolution of the disease. Raman spectroscopy is a powerful technique for analyzing the constituent molecules and structures on a micrometric scale, and its high resolution makes it possible to study local changes in biological tissues. Vibrational spectra of bone tissues can be obtained by Raman spectroscopy also in situ, using optical fibers, which is a non-destructive approach simultaneously providing information about mineral and organic matrix of bone.

The advantages of Raman spectroscopy are that it can be applied to specimens of any size, from centimeters to microns, with limited influence from the presence of water, and it can accommodate micron-scale mechanical deformation. Taking advantage of the characteristics of Raman spectroscopy, previous researches could also be conducted on the role of bone minerals in biomechanics and bone fractures [15] and the changes in the spectra of Raman bands related to the O-H stretching of water by Raman spectroscopy and correlated them with the changes in ion concentrations in intracellular and extracellular fluids [16]. 

In recent years, research on the process and causes of various bone diseases has begun to use advanced Raman spectroscopy methods. Although some studies [17] claim that the molecular composition of the necrotic region of the femoral head is the same as that of the healthy region, the improved performance of Raman spectrometers has made it possible to perform XY imaging measurements and to obtain continuously and averaged Raman spectra over small tissue areas, where the Raman spectra of the necrotic and healthy regions appear different. However, since the sample bone is heterogeneous, single-point Raman spectroscopy cannot adequately represent bone’s overall chemical fine structure without a statistical approach. Therefore, spatial information is required. For this reason, Raman spectroscopic imaging is particularly useful for the analysis of large portions of intricately organized systems such as bones and teeth [18].

This study investigates the changes in the molecular composition of the region of ONFH in alcoholic and steroidal ONFH and the spectroscopic structure of healthy and osteonecrotic bone tissue to search for specific fingerprints related to the structure of bone apatite or collagen and caused by the condition. Moreover, the study focused on specific spectroscopic characteristics that can be associated with osteonecrosis with different origins, alcoholism, or the use of steroids.

The aim of this methodological study, performed on a limited number of specimens, is to show a pilot study and offer directions for further studies on the use of Raman spectroscopy as a fast-screening complementary diagnostic tool for bone-related diseases.

## 2. Materials and Methods

### 2.1. Information on ONFH Samples

We received femoral head specimens of the osteonecrotic femoral head from Prof. Nobuhiko Sugano, Department of Medicine, Osaka University Graduate School of Medicine. The status of osteonecrosis was assessed by the institution, using histological results that are not included in this manuscript. To reduce the possible sources of variability, the bone density of all patients was checked and osteoporotic patients were excluded from the cohort. We conducted Raman spectroscopy studies on a total of seven specimens: three femoral heads from alcoholic ONFH and four femoral heads from steroidal ONFH. The 7 detailed patient information for the 7 specimens is shown in Table 1.

It must be noted that since all patients with alcohol-related osteonecrosis were young/middle-aged males and all patients with steroid-related osteonecrosis were relatively older females it was impossible to rule out completely the effects of gender and age on the spectroscopic results. Based on previous literature references and scientific consensus, due to inter-sex differences in structural properties, the healthy bone of elderly women is expected to have, on average, a lower bone quality when compared to younger men [19,20], and in particular lower density and mineralization.

**Figure 1:**
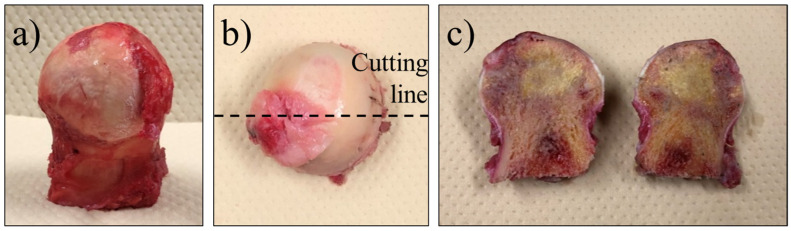
Preparation of the samples: (**a**) an anterior view of the femoral head removed after surgery, (**b**) cutting line, (**c**) front cross-section.

The femoral heads were surgically extracted from the patients, as shown in Figure 1a. The femoral head was then cut in half, front to back, as shown in Figure 1b, and stored in a cold chamber (−8 °C) environment to prevent spoilage. The samples were then removed from the storage and analyzed while being kept cold by using dry ice, to ensure that they remained fresh. The cut surface of a femoral head sample is shown in Figure 1c. In some cases, the necrotic region could be visually identified by a color change and bone density status, but in most cases, it was marked by the surgeon after removal and histological testing. 

Due to the limited number of specimens, in order to minimize the effects of age, gender, and other health conditions on the results, spectroscopic experiments were conducted by Raman spectroscopy on dependent triplets of areas exploiting pairing of patient data in healthy and diseased areas. For each specimen (femoral head), three regions were investigated: the center of the osteonecrotic region, a healthy portion of the femoral head, and the “boundary region”, defined as a point in which the signs of osteonecrosis start to appear, by optical observation.

### 2.2. Measurement Method by Raman Spectroscopy

The Laser Raman Microscope RAMANtouch (Nanophoton, Osaka, Japan) is a Raman spectrometer optimized for Raman imaging of surfaces. The samples can be observed both by conventional optical microscopy and by Raman imaging, in the latter case by using an array of 400 detectors to obtain a sequence of linear scans that are then used to produce a Raman image that can be overlapped with their correspondent optical micrograph and have a sub-micrometric lateral resolution.

The software is operated by using two dedicated software, The RAMAN Imager2 control software and RAMAN Viewer (Nanophoton, Osaka, Japan).

After checking the output of three different lasers (green, blue, and near-infrared), we adopted a near-infrared (NIR) laser with a wavelength of 785.13 nm for the analysis. The laser power was set to 49.11 mW and the central wavenumber of the monochromator was set to 1350.00 cm^−1^. The exposure time (/line) was 20 s and the number of integration was 4 times. 

The objective lens was a TU Plan Fluor 20×/ NA 0.45, and the measurement mode was XY Imaging with a vertical × horizontal area of 50.9 μm × 415.47 μm. This laser Raman microscopy technique is capable to provide a large number of spectra, that can be then used to obtain averages at different locations, depending on the condition of the bone tissue. Areas were labeled depending on their condition as “necrotic”, “healthy” and “boundary”.

In particular, this research makes use of the bands located at 960 cm^−1^ (for the hydroxyapatite), 1240–1270 cm^−1^ (for the Amide III), 1450 cm^−1^ (for the Amide II), and 1660 cm^−1^ for the Amide I). For each band, intensity and the full width half maximum (FWHM) were recorded. The FWHM of the Raman band at 960 cm^−1^ is related to the complex off-stoichiometric change in the columnar Ca structure of bone hydroxyapatite and asymmetric phosphate band at 960 cm^−1^. The morphological changes of Raman bands belonging to the symmetric phosphate stretching (ν_1_) band at 960 cm^−1^ correspond to the Raman bands [21]. The FWHM of the Raman band at 960 cm^−1^ is often used as an indicator of the crystallinity of Hap. The degree of crystallinity of the apatite crystal is considered to increase with the reciprocal of the FWHM [22]. As the FWHW increases, the degree of crystallinity decreases. This means that the crystallite size is reduced and the crystal structure is altered.

It has been reported that the crystallinity of Hap also increases with the age of the tissue [23]. This increase is due to the deterioration of structural and tissue-level mechanical properties with age.

In this experiment, we used the value of the FWHM to estimate the degree of crystallinity. The intensity of the 960 cm^−1^ band, on the other hand, is universally accepted as an indicator of bone mineral content. There is disagreement about which brand to use as an indicator of matrix content [17], but it is also recognized that collagen quality plays a fundamental role in the process of bone embrittlement [15], so the ability to extract information on collagen from Raman spectroscopy is crucial.

Candidates for bands to be used as indicators include amide I (1660 cm^−1^) [22,23], amide III C-N-H stretch (1243–1269 cm^−1^) [22,23], and CH_2_ deformation (1450 cm^−1^) [24,25]. It has been reported that the intensity ratio of phosphate/amide III is proportional to the calcium content and is described as mineralization [21]. The measured areas of important marker bands such as phosphate ν1 (960 cm^−1^), mono-hydrogen phosphate ν1 (1003 cm^−1^), type B carbonate ν1 (about 1070 cm^−1^), collagen CH_2_ twisting [26] (about 1450 cm^−1^), and collagen amide I C=O stretch [26] (about 1660 cm^−1^) indicate that the phosphate/matrix ratio. Some studies have calculated the phosphate/matrix ratio [27], phosphate/hydrogen phosphate ratio, and carbonate/hydrogen phosphate ratio from the measured area of important marker bands such as collagen CH_2_ [28,29]. It was used as a denominator in the introduced optical coefficients (k) [30]:

$$k=\frac{I_{i}}{I_{1660}}$$

*I_i_* is the intensity value at the wavenumber of the analyzed components [31,32].

The ratio I960cm^−1^/I1660cm^−1^ determines the degree of leaching of mineral components in the process of demineralization [29]. 

In the present study, we calculated the intensity ratios I960cm^−1^/I1243–1269cm^−1^, I960cm^−1^/I1450cm^−1^, and I960cm^−1^/I1660cm^−1^ in the necrotic, boundary, and healthy regions of each sample and analyzed their relationships with the health status of the tissues.

As previously stated, the experiments were conducted on triplets of dependent areas and exploiting the pairing of patient data in healthy and diseased areas. The quality of the bone tissue in the osteonecrotic areas was considered with respect to the surrounding healthy bone tissue. This limits the diagnostic capability of the method, meaning that it can only accurately evaluate osteonecrosis upon a comparison with autologous tissue. In order to generalize the method to a standard one for accurate and patient-dependent diagnoses, further investigations and statistics are needed that take into account additional factors such as age, gender, and eventual concurrent pathologies.

## 3. Results

Measurement in XY (2D) imaging mode enables the observation of the planar distribution of the molecular structure of the sample using linear arrays of detectors. By selecting a rectangular area of the sample on the microscope monitor and specifying the number of scans, a line laser beam is scanned to cover the whole area of the sample, making it possible to acquire thousands of spectra in only a few hours of measurement.

**Figure 2:**
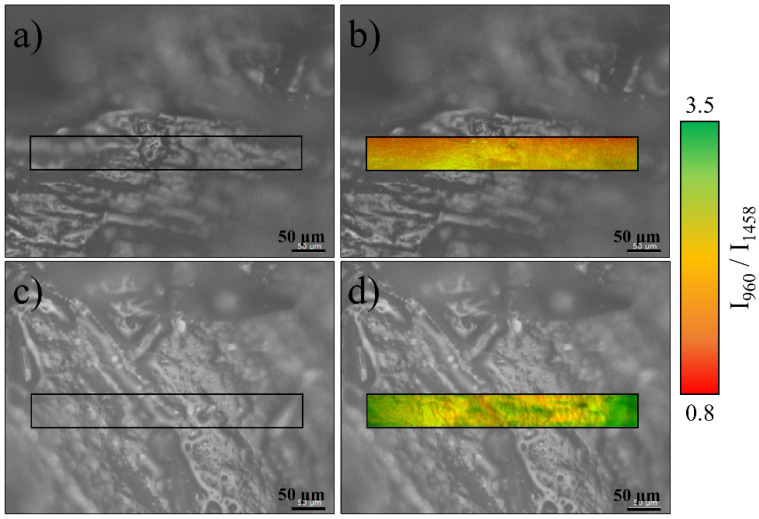
Optical and Raman imaging of the surface of Sample No7: (**a**) optical image of an osteonecrotic area, (**b**) Raman imaging of the same area, (**c**) optical image of a healthy area, (**d**) Raman imaging of the same area.

To obtain a Raman imaging map, specific bands are selected from a representative spectrum and their intensity or ratio is expressed using a color scale on the whole area. Figure 2 shows the distribution of the ratio between hydroxyapatite and collagen-related bands. The phosphate vibration at around 960 cm^−1^ is related to the ν_1_ PO_4_ band for hydroxyapatite, which is the strongest marker for the bone mineral fractions. A relatively strong Amide III band can be found at about 1256 cm^−1^, δ(CH, CH_2_), and proteins + lipids also possess a relatively intense band around 1458 cm^−1^, and Amide I showing a band around 1677 cm^−1^. All three bands have a similar intensity distribution, being almost proportional to each other on the investigated area. For the purpose of Figure 2, the Amide II band located around 1458 cm^−1^ is shown as the denominator.

Micrographs (a) and (c) show two locations of sample No. 7 steroidal osteonecrosis of the femoral head. The (a) area is related to bone tissue with clear signs of osteonecrosis, while the (c) area is located in a relatively healthy region of the same sample. Raman imaging taken on the same regions are presented in (b) and (d). It can be observed that in the region (b) the intensity of the Amide III-related band is stronger, while the band related to hydroxyapatite is weaker and preferentially located in the bottom part of the region. For the healthy bone tissue of region (d), the hydroxyapatite signal is up to 3.5 stronger than that of Amide III, and it is mainly concentrated on the central region of the image. 

**Figure 3:**
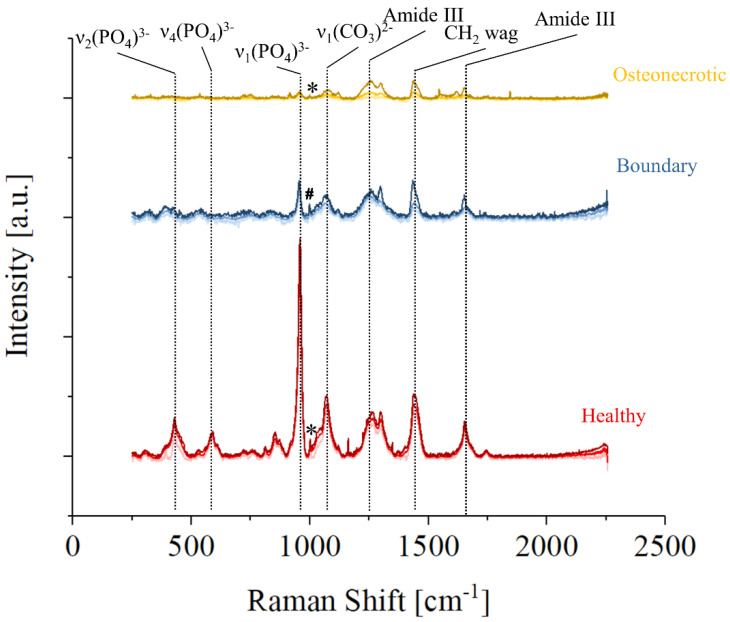
Representative Raman spectra of three different regions of Sample No7: healthy in red, the boundary in blue, and osteonecrotic in yellow. For each set of data, the average spectra and the limits of the 90% confidence interval are presented in different tones. Phenylalanine is marked with an “*” and an unknown band, marked with a “#”, appears at slightly lower Raman Shifts.

Figure 3 shows the average Raman spectra collected by Raman imaging in three regions of sample No. 7, healthy (red), osteonecrotic (yellow), and boundary (blue), defined as the region in which osteonecrotic tissue starts to appear by optical observation. For each set, the upper and lower lines are the limits of the 90% confidence interval, while the line in the middle is the average spectra in that area of the sample. It can be observed that the overall spectral intensity decreases moving from healthy to the osteonecrotic bone, while the relative intensity of specific regions of the Raman spectra varies greatly depending on the region. Band related to hydroxyapatite vibrations are barely detectable on the average spectra of the necrotic tissue of Figure 3, due to a lack of intensity coupled with a progressive increase in the FWHM of the band. Amides and the CH_2_ wagging vibrations, on the other hand, are still clearly observable. It can be speculated that the osteonecrotic tissue has a lower amount of mineral bone apatite when compared to the surrounding tissue, but it must be noted that the intensity of the band is also associated with the degree of order in the crystallographic structure: disordered hydroxyapatite crystals produce less intense vibrational bands with larger FWHM. 

While the strongest Raman vibration of the osteonecrotic tissue appears to be the Amide III band situated at about 1260 cm^−1^, the average spectrum collected in the boundary tissue shows a relatively stronger CH_2_ wagging band. Considering the whole region from 1000 to 1750 cm^−1^ and associated with collagen and proteins, the strongest Raman intensity was recorded in the boundary regions, which seems to indicate that collagenous tissue is preferentially formed in those areas.

A small peak associated with Phenylalanine and marked with an “*” could be clearly observed in the healthy and osteonecrotic bone, but not in the boundary region where an unknown intense band, marked with a “#”, appears at slightly lower Raman Shifts.

**Figure 4:**
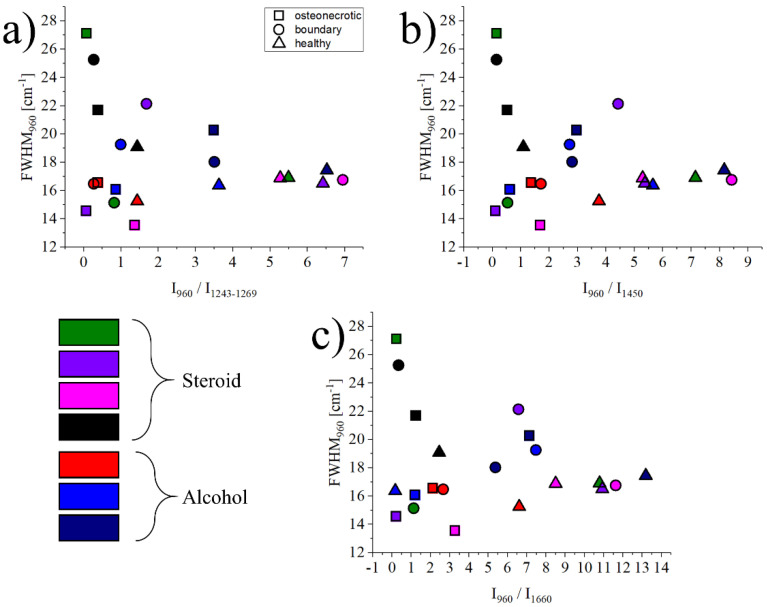
Graphs showing the relationship between the intensity ratios (I960cm^−1^/I1243–1269cm^−1^, I960cm^−1^/I1450cm^−1^ and I960cm^−1^/I1660cm^−1^) and the FWHM of the band located at 960 cm^−1^ for the healthy, boundary and ON regions of all samples (**a**–**c**). Symbols and colors are as follows: △ healthy, □ osteonecrosis and ○ boundary tissue; **green**, **purple**, **pink** and **black** for steroid, **red, blue** and **dark blue** for alcohol.

The graph in Figure 4 shows the relationship between the FWHM of the band at 960 cm^−^^1^ and related to ν_1_ (PO_4_)^3-^ vibrations in hydroxyapatite, and the intensity ratio between the bands at 960 cm^−1^ vs. three regions: 1243–1269 cm^−1^, representing Amide III vibrations, 1450 cm^−1^ representing CH_2_ wagging, and 1660 cm^−1^ for Amide I vibrations. Necrotic tissues are marked with a circle, boundary regions with a square, and healthy bone references with a triangle. Each symbol represents the average of about 1500 spectra, with the statistical dispersion omitted for clarity. For reference, the dispersion for necrotic tissue is in usually the range of ±22% of the average value, and it reduces to about ±10% for the healthy tissue. Even if the data is highly scattered, which is a common occurrence when analyzing biological samples coming from different donors, osteonecrotic tissues tend to have a lower ratio (x-axis) and a more scattered FWHM (y-axis) when compared to their healthy counterparts.

**Figure 5:**
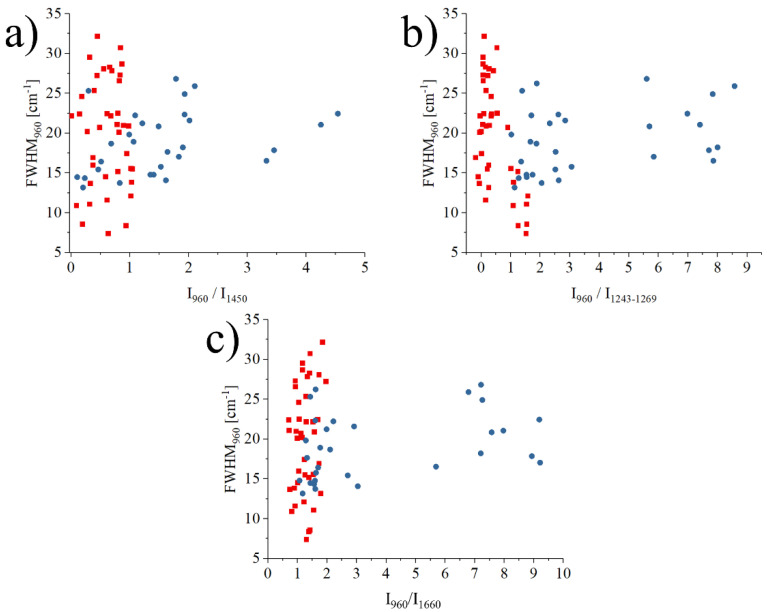
Graphs (**a**–**c**) of the relationship between the each of intensity ratio I960cm^−1^/I1450cm^−1^, I960cm^−1^/I1243–1269cm^−1^, I960cm^−1^/I1660cm^−1^ and FWHM of the band located at 960 cm^−1^. Different trends between alcoholic necrosis and steroidal necrosis regions are shown.

Figure 5 investigates the correlations between the FWHM and the intensity ratio I960cm^−1^/I1243–1269cm^−1^, the FWHM and intensity ratio I960cm^−1^/I1450cm^−1^, and the FWHM and intensity ratio I960cm^−1^/I1660cm^−1^ for the data measured only in the necrotic region of all samples. In each graph, a single data point represents the average of 11 single point spectra (11 × 11) on an osteonecrotic area of 100 μm^2^. These three correlations represent the relationship between the crystallinity of the mineral bone apatite and the ratio between mineral and matrix (collagen). The blue points in the graph represent the necrotic area data of alcoholic ONFH, and the red points represent the necrotic area data of steroidal ONFH. 

What was more noteworthy was that the regions on the graph where the data for the alcoholic femoral head necrosis region and the steroidal femoral head followed a diver-gent distribution, even if the difference resulted to be not statistically significant. This suggested that the bone components analyzed at the molecular level in the region of ONFH could show differences between alcoholic and steroidal osteonecrosis of the femoral head, at a spectroscopic level.

Considering both the barycenter of the spread distribution and the mode, data points related to osteonecrosis associated with alcoholism are usually associated with higher ratios of mineral to amide III (1243–1269 cm^−1^), mineral to collagen (1450 cm^−1^), and mineral to amide I (1660 cm^−1^) than osteonecrosis in steroidal ONFH. On the other hand, steroidal ONFH has a tendency towards lower crystallinity with lower values of the ratio of minerals to each organic matter in the matrix, compared to osteonecrosis of alcoholic ONFH. As these results are not normalized by gender and all patients with steroid osteonecrosis are female, it is also possible that what we are observing are the effects of early-stage osteoporosis that has yet to be diagnosed by conventional methods. If we rule out osteoporosis, a hypothesis that needs to be confirmed in future studies but seems unlikely considering the high crystallinity of the hydroxyapatite, it can be concluded that the mechanism of osteonecrosis was different between alcoholic and steroidal ONFH and Raman spectroscopy is a promising tool for further investigations.

**Figure 6:**
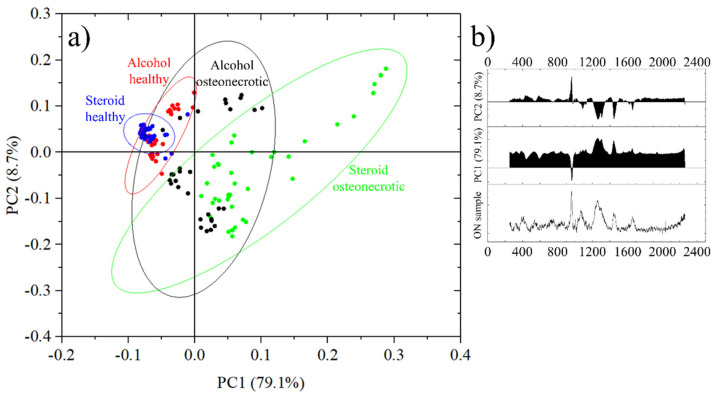
(**a**) Principal Components Analysis of the different samples, divided into four groups depending on the origin and health status and (**b**) Principal components PC1 and PC2 with their score and an osteonecrotic spectrum for comparison.

The results of the PCA analysis are resumed in Figure 6a. Each data point represents the average spectra of an 11 × 11 points Raman map acquired on a 10 μm × 10 μm region. Healthy bone from steroid-osteonectrotic patients is marked by blue circles, healthy bone of alcohol osteonecrotic patients in red and the correspondent osteonecrotic tissue in green and black, respectively. For each group of samples, the circled areas represent the 95% confidence ellipses. It can be seen that all most all blue and red dots are in quadrant II, while green circles are distributed in quadrants I and IV and black circles can be observed in quadrants I, III and IV. 

In this representation, in Figure 6b PC1 has a score of 79.1% and increases with the overall fluorescence as opposed to the intensity of the band at 960 cm^−1^ and related to the ν_1_ (PO_4_)^3−^ vibrations in hydroxyapatite.

PC2 has a score of just 8.7% and it grows with the intensity of the band at 960 cm^−1^ versus the other bands related to Amides and CH_2_ wagging vibrations. PC1 + PC2 account for a total score of 87.8%. Other principal components had a score of less than 2.0% and were therefore excluded from the representation.

Taking into account the roles of PC1 and PC2, the circles in the first two quadrants, I and II, represent areas of the sample with a good degree of mineralization, while the samples in quadrants III and IV show more limited degrees of mineralization.

On the other hand, samples in quadrants II and III have a low level of fluorescence while samples in quadrants I and IV have higher levels of fluorescence.

From Figure 6a we can observe that the healthy bone tissue of steroid osteonecrotic individuals has a low fluorescence, a good degree of mineralization and a small dispersion of the data (smaller confidence ellipse). Similar results were observed for osteonecrotic alcoholic patients, but with a larger statistical dispersion and a bi-modal distribution, related mainly to the degree of mineralization.

For alcohol osteonecrotic tissue, the bi-modal distribution seems to be confirmed, with either good mineralization but high fluorescence, or poor mineralization and lower fluorescence. The worst scores, from the point of view of data reliability, were obtained from steroid-osteonecrotic tissue where the fluorescence was always high and the mineralization randomly distributed, but with the statistical mode in quadrant IV.

## 4. Discussion

ONFH is a disease in which the femoral head caves in due to impaired blood circulation, resulting in the loss of both load and wear bearing functions. This disease is classified as idiopathic ONFH if there is no obvious underlying disease, and is further divided into steroidal, alcoholic, and idiopathic in a narrower sense. On the other hand, if there is an underlying disease with a clear causal relationship to the occurrence of necrosis, such as trauma, embolism, or irradiation, it is classified as symptomatic ONFH. As ONFH can have many different risk factors, it is possible to speculate that the disease also proceeds at a different pace, resulting in morphological and crystallographic features appearing and disappearing in the relative Raman spectra.

There are various theories on the mechanism of ONFH, including fat embolism, increased intramedullary pressure, blood coagulation abnormalities, vascular lesions, and micro-fractures. In particular, the details of the mechanism of ONFH head associated with systemic steroid administration and alcohol intake have not been clarified. For asymptomatic ONFH, the relationship between symptom onset and alcohol was reported. And it has been reported that the most important prognostic factor is the location and size of the lesion and that the collapse of the femoral head is extensively progressive [32,33]. Studies have reported that the habit of drinking large amounts of alcohol is a high risk for symptomatic progression of asymptomatic ONFH [34]. In addition, in a study that evaluated the proportion of patients with ONFH due to a history of systemic steroid administration and habitual alcohol intake using two different questionnaire methods, it was suggested that the definition of steroidal ONFH should include information on the dose and duration of steroid administration and that a small number of steroids should be used to treat It has been demonstrated that even small amounts of steroids can cause ONFH [35]. Furthermore, it has been shown that reduction of oxidative stress is effective in steroid-induced ONFH [36]. It has been reported that Raman spectroscopy may be useful in protein structural studies by identifying characteristic bands that indicate molecular conformation and hydrogen bonding [37]. In the field of biomechanical research, Raman spectroscopy has been previously used to study embrittlement after irradiation using a mouse tibia model [38]. Regarding bone and calcium phosphate biomaterials, previous Raman studies were also used to monitor their behavior over a period of 8 months [39]. Concerning ONFH, the exact effect of material effects (crystallinity, phosphate content, phosphate/matrix ratio, strength ratio of each organic material, etc.) on ONFH is still not fully understood [40].

In this study, we measured and analyzed the bone components by Raman spectroscopy, in order to investigate the effects of osteonecrosis on a molecular level. As a result, we found that there were differences in the bone composition and crystallinity at the necrotic area of the femoral head when compared to the surrounding, healthy tissue. Furthermore, we identified spectral differences that might be related to the cause of necrosis, being it alcoholism or steroids. It must be noted that as the cohort for steroid use was female, it is difficult to rule out completely osteoporosis as a possible contributing cause of the spectroscopic differences, but Figure 6 shows no statistically significant spectroscopic difference between the two types of healthy tissue, making the presence of osteoporosis unlikely [21]. Moreover, the barycenter of the scatter plots for alcohol healthy tissues is at slightly higher PC1 values than the one related to steroids.

The relationship between the degree of crystallinity of HAp and the Mineral-to-Matrix intensity ratio (intensity of Raman band of HAp/intensity of each organic substance) was analyzed using Raman spectroscopy in the necrotic, borderline, and healthy regions of the femoral head, suggesting that the process of change in bone components may be different between alcoholic and steroidal osteonecrosis. This result suggests that alcoholic and steroidal compounds may undergo different processes in changing bone composition.

One possible explanation for the different spectroscopic responses relies upon the mechanisms that cause osteonecrosis. Alcohol causes hyperlipidemia, a condition that causes an excess of fatty cells in the body. The increased fatty content in the bloodstream, in the form of triglycerides, but also low- and very-low-density lipoproteins (LDL and VLDL) can cause obstructions in the blood vessels [41]. Due to its relatively low levels of vascularization, the head of the femur is especially susceptible to decreased blood flow, resulting in aseptic necrosis. Moreover, adipocytes also tend to accumulate in the femur head, resulting in an increase in blood pressure which further reduces blood circulation. Necrotic cells are then replaced by fibrotic tissues [42].

From a spectroscopic point of view, the newly formed fibrotic tissue would have relatively intense Raman vibrations in the region between 1200 and 1750 cm^−1^, corresponding to Amides, as observed in the osteonecrotic spectrum of Figure 3. These regions correspond to the black dots on the third and fourth quadrant of Figure 6. It is worth noting that about 60% of the “healthy” alcoholic bone tissue has a PC2 score lower than the steroid healthy tissue average, suggesting a possible general lack of mineralization, but the difference between the two sets of data is not statistically significant.

Steroid-induced osteonecrosis is also related to the accumulation of fat, in particular in the subchondral vessels [43]. Steroid induces osteonecrosis often proceeds much faster than the correspective alcohol-induced [44], and this might result in a lower amount of fibrotic tissue formed in the region. It was also reported that TNF-a mediated inflammatory macrophage polarization might contribute to the pathogenesis of steroid-induced osteonecrosis [45].

In Figure 6, data points related to steroid-induced osteonecrosis have, on average, higher PC1 scores, meaning that they are affected by higher levels of fluorescence, which have been previously associated with damaged apatite and collagen and decayed teeth [21,46].

The results of this study may have implications for orthopedic surgeons treating ONFH. Although Raman spectroscopy has been used to investigate changes in bone composition associated with ONFH, we used Raman spectroscopy to investigate the difference in crystallinity of HAp in the necrotic region of alcoholic and steroidal ONFH and the difference in the intensity ratio (intensity of Raman band of HAp/intensity of each organic material) in the necrotic regions of osteonecrosis and steroidal ONFH. 

We also focused on the difference in crystallinity of HAp and the difference in intensity ratio (intensity of Raman band of HAp/intensity of each organic material) in the necrotic region of osteonecrosis, and no previous study mentions the difference in the mechanism of occurrence. For future research, it would be necessary to increase the number of measurement samples and thus improve the statistical reliability and validity. It would also be necessary to clarify the risk factors of the difference in bone composition between alcoholic and steroidal ONFH. Further investigations on the interpretation and relationship of each variable obtained from these Raman spectra is essential to, ultimately, enable the assessment of bone strength in clinical practice and in real-time [47], the advancement of technology for the evaluation of morphological and physical material properties of bone performed non-invasively in humans is urgent.

Previous investigations proved that Raman spectroscopy can detect alterations in the chemical structure of the bone two major components, hydroxyapatite crystals and collagen matrix [30,31,32]. Building upon these findings, spectroscopic methods were made available for the detection of bone structural changes related to osteoporosis [21]. By exploiting additional data related to osteoporosis, Raman spectroscopy might prove complementary as a diagnostic tool for the simultaneous detection of multiple bone pathologies.

In order to confirm the diagnostic capabilities of Raman spectroscopy and to extract statistically validated calibration algorithms for the specific detection of each bone-related disease, a more extensive, multi-centric study will be necessary in the future.

## 5. Conclusions

This pilot study, conducted on a limited number of patients, demonstrates that Raman spectroscopy is a useful and powerful tool for the analysis of bone quality and for the diagnosis of bone-related diseases, in particular associated with osteonecrosis. 

In the case of steroid-induced osteonecrosis, the principal component analysis proved to be able to discriminate between healthy and osteonecrotic tissue, with high accuracy.

In the case of alcohol-induced osteonecrosis, the was a certain degree of overlapping in the PCA graph and thus a lower accuracy, but osteonecrotic tissue resulted in either higher values of PC1 or lower values of PC2, in most of the cases, with a bimodal distribution.

When comparing the two types of osteonecrotic tissue, results were mostly overlapped, but steroid-induced osteonecrotic tissue reached higher PC1 values due to the higher background fluorescence generated.

Differences between the healthy tissues resulted to be statistically not relevant and probably related to the biases in the selection of the specimen.

## Figures and Tables

**Table 1:** Femoral heads information with osteonecrosis. ※ ON: Osteonecrosis.

Sample Number	Surgery Date	Medical Condition	Aetiology	Side	Age	Gender
1	14 February 2020	ON※	Alcohol	Left	28	Male
2	27 March 2020	ON※	Steroid	Left	60	Female
3	29 May 2020	ON※	Alcohol	Left	37	Male
4	3 June 2020	ON※	Alcohol	Left	51	Male
5	4 June 2020	ON※	Steroid	Left	59	Female
6	26 June 2020	ON※	Steroid	Left	56	Female
7	2 October 2020	ON※	Steroid	Right	73	Female

## Data Availability

Data available on request due to privacy restrictions.
